# Changing forms of HIV-related stigma along the HIV care and treatment continuum in sub-Saharan Africa: a temporal analysis

**DOI:** 10.1136/sextrans-2016-052975

**Published:** 2017-07-23

**Authors:** O Bonnington, J Wamoyi, W Ddaaki, D Bukenya, K Ondenge, M Skovdal, J Renju, M Moshabela, A Wringe

**Affiliations:** 1 Department of Epidemiology and Population Health, London School of Hygiene & Tropical Medicine, London, UK; 2 National Institute for Medical Research, Mwanza, Tanzania, United Republic of; 3 Rakai Health Sciences Program, Rakai, Uganda; 4 Medical Research Council/Uganda Virus Research Institute Research Unit on AIDS, Entebbe, Uganda; 5 Kenya Medical Research Institute (KEMRI), Nairobi, Kenya; 6 University of Copenhagen, Copenhagen, Denmark; 7 Biomedical Research and Training Institute, Harare, Zimbabwe; 8 Malawi Epidemiology and Intervention Research Unit, Karonga, Malawi; 9 Africa Health Research Institute, KwaZulu Natal, South Africa; 10 University of KwaZulu Natal, South Africa

**Keywords:** AIDS, QUALITATIVE RESEARCH, AFRICA, HIV

## Abstract

**Objectives:**

Stigma remains pervasive for people living with HIV (PLHIV) in sub-Saharan Africa, undermining care engagement. Using *everyday*, *biographical* and *epochal* temporalities, we explored the manifestation of stigma at different stages of the HIV care continuum in seven health and demographic surveillance sites in Eastern and Southern Africa.

**Methods:**

Between 2015 and 2016, we conducted qualitative in-depth interviews with 264 PLHIV, 54 health providers and 48 family members of people who had died from HIV. Topic guides explored experiences of HIV testing, care and treatment services. Data were analysed thematically, aided by NVivo 10.

**Results:**

In *everyday* time across these communities, stigma was evident in the *presence* of gossiping and the relative *absence* of supportive interpersonal discourse, which fuelled judicious disclosure. This was especially disruptive at testing, counselling and early antiretroviral therapy adherence stages of care. *Biographical* time framed everyday stigma events, highlighting the dilemma of disclosure in relation to sexual relationship norms, as well as the interfacing of age and healthcare continuum points. *Epochal* patriarchal relations gave a structural context to everyday and biographical stigma dynamics. Historical shifts to social acceptance of PLHIV within these communities, while positive, were complicated by stigma in everyday life and in respect of biographical goals like having a family. Moreover, low community-level resistance to HIV-related stigma jeopardised stigma reduction strategies.

**Conclusions:**

Despite improvements to HIV care services, stigma remains pervasive across the HIV care continuum in these sites. Context-specific interventions are needed to address stigma and discrimination of PLHIV within the community and in health services, and greater reflection is required to ensure policies aiming to expand HIV treatment do not exacerbate stigma and result in negative HIV outcomes.

## Introduction

Stigmatisation involves individual, cultural and structural processes which, through the use of power, combine to produce negatively valued difference.^1^ HIV-related stigma may hinder attempts to eliminate AIDS by 2030.^2^ Therefore, the 2016 UN General Assembly Political Declaration on Ending AIDS has set out a target of eliminating HIV-related stigma and discrimination by 2020. This ambitious target follows earlier hopes that antiretroviral therapy (ART) roll-out would help reduce stigma.^3^ ^4^ However, public stigma—negative attitudes and beliefs that the general public hold towards people living with HIV (PLHIV)—remains a significant challenge across Africa.^5^ ^6^ Moreover, ART may have mixed effects on stigma reduction, making the UN AIDS target problematic. For instance, Roura *et al*^7^ noted the emergence of a stigma paradox 2 years after ART availability in rural Tanzania. On the one hand, PLHIV were seen as less burdensome by themselves and others, leading to decreased self-stigma (ie, internalising public stigma) and increased social support, which helped normalise HIV and increased voluntary counselling and testing (VCT) uptake. This is supported by other studies from sub-Saharan Africa in respect of decreasing public stigma^8^ and increasing the coping skills of PLHIV.^9^ On the other, new sources of stigma emerged whereby fears of sexual transmission were exacerbated, fuelling moral outrage, and a persistence in blame for being ‘irresponsible’ in spreading the virus through sexual activity abounded. This, in turn, led to widespread anticipated stigma (ie, fear of being stigmatised), othering and a collective denial that reduced VCT uptake.

Most qualitative research exploring the relationship between stigma and accessing HIV services has focused on how it influences HIV testing uptake.^7^ ^10–12^ Few studies have explored the effect of stigma on PLHIV's engagement with HIV services once diagnosed and expected to initiate ART, and adhere to lifelong pill-taking and clinic visits.^13^ ^14^ Additionally, most stigma studies have been quantitative rather than qualitative,^11^ ^15^ ^16^ often looking at public stigma^5^ ^6^ rather than experienced stigma (ie, the actual experience of discrimination or prejudice) and anticipated stigma among PLHIV. Furthermore, stigma studies in the context of ART roll-out have tended to focus on one country.^7^ ^11^ ^15^ ^17^ However, if HIV-related stigma is going to be reduced, there is a need to understand how stigma manifests and is experienced and anticipated at different stages of HIV care. Moreover, it is imperative that this is understood across the health service and policy contexts of different epidemics. Understanding the geographical and care continuum manifestations of stigma enables antistigma interventions to be more efficiently targeted.

### Three overlapping stigma temporalities

Essential to this is having a nuanced understanding of the temporality of stigma events. Stigma studies focusing on the effect of ART on VCT stages tend to have a singular view of time (ie, either before and after new treatment, or the point in time when testing occurs), rather than one where overlapping modalities of time effect the contexts and concerns of PLHIV. In contrast, this paper uses three perspectives on time: *everyday*, *biographical* and *epochal*.^18^ These are not *philosophically* discrete, detached ways within which stigma dynamics play out, but imbricated ones that flow into and influence each other. An *analytical* understanding of the role of time—in combination with social and cultural structures and human agency—provides clues for antistigma practices.

*Everyday* time concerns the description, immediacy and repetition of daily stigma events. Some questions PLHIV might navigate in everyday time are: How is HIV discussed day-to-day in public spaces? Given what others know about me, will I experience stigma today if I socialise with friends?

*Biographical* time is slightly different to the quotidian repetition of everyday stigma. Pertinent questions here include: When should people get tested for HIV during their lifetimes? When and to whom should they disclose their status? Should they start treatment when advised? The key question cutting through all of these is ‘What is at stake within this and future biographical moments—will my life chances and projects be limited in some way if I am associated with HIV’?

*Epochal* or historical time relates to whether conditions influencing HIV-related stigma are stable and enduring, or elaborated and transformed. So, for instance, is gossiping about PLHIV a practice that is being observed less nowadays than in the past? Are communities unified and enabled to challenge stigma, or is stigma resistance still sporadic and individualised? How have developments in treatment availability and coverage influenced the level and nature of stigmatising interactions?

Using these temporalities, we aim to investigate how HIV-related stigma manifests in PLHIV's accounts of their HIV care across seven rural locations in six sub-Saharan African countries and how these experiences shape their treatment engagement.

## Methods

### Settings and sample

This paper draws upon qualitative data from in-depth interviews conducted in a multicountry study (the ‘Bottlenecks’ study) exploring the interaction of PLHIV and HIV care services. Additional methodological details concerning the Bottlenecks study can be found in the online methods supplement found within the editorial http://dx.doi.org/10.1136/sextrans-2017-053172.^19^ Ethical approval was obtained from London School of Hygiene & Tropical Medicine (LSHTM) (Ref: 10389) and by local ethics boards at the study locations. Informed and written consent was obtained from all participants. The study took place in Karonga (Malawi), Rakai and Kyamulibwa (Uganda), Kisesa (Tanzania), Kisumu (Kenya), Manicaland (Zimbabwe) and uMkhanyakude (South Africa). Through health and demographic surveillance site (HDSS) datasets and ART clinics, we purposively sampled 264 PLHIV and 48 family members of deceased people with HIV to achieve a broad distribution of sex, age and care history. Family members were identified through HDSS verbal autopsy reports. Additionally, 54 healthcare workers were purposively sampled across the sites to ensure a range of perspectives on the provision of HIV services from different cadres (see table 1). Further information can be found in the online supplementary file.

**Table 1:** Description of study participants by site

			Person living with HIV			
Country	HDSS	HCW	Never ART	On ART	LTFU†	Family member of deceased
Uganda	Rakai	6	15	15	6	8
Uganda	Kyamulibwa	5	8	16	4	5
Kenya	Kisumu	8	10	15	6	11
Tanzania	Kisesa	7	13	14	4	6
Malawi	Karonga	5	9	27	4	6
Zimbabwe	Manicaland	4	16	35	8	6
South Africa	uMkhanyakude	19	16	17	6	6
	Total	54	87	139	38	48

†Lost to follow up from a HIV clinic for >90 days;

ART, antiretroviral therapy; HCW, healthcare Worker; HDSS, health and demographic surveillance sites; LTFU, lost to follow up.

### Data collection and analysis

Fieldworkers were trained in each location. They administered a short screening tool before conducting each in-depth interview to ensure participant eligibility and to obtain diagnosis and treatment history of PLHIV. Face-to-face in-depth interviews, conducted in local languages, were chosen owing to the sensitive and personal nature of information being discussed. Topic guides for PLHIV explored experiences of interactions with healthcare services throughout the care continuum, the salience of community health issues and HIV-related stigma. Some repeat interviews were done to build rapport and discuss more sensitive issues. Interviews with family members of the deceased focused on the circumstances that contributed to their loved ones' deaths. Healthcare provider interviews solicited reflections on engaging PLHIV and delivering HIV services. Details of how these topics were generated can be found in the online-only supplement. Interviews lasted approximately 45–90 min and took place in participants' homes or private clinic areas. Data were collected between October 2015 and April 2016. Interviews were audio-recorded, then anonymised, and either summarised into detailed reports (Kyamulibwa) or transcribed (Karonga, Kisesa, Rakai, Kisumu, Manicaland, uMkhanyakude) and translated into English. Summaries were done in Kyamulibwa due to researchers at this site being historically trained in this method. All data were stored in secure password-protected locations. Participants were recruited until thematic saturation was reached.

A latent thematic analysis was conducted, which entails a process of identifying and analysing patterns within qualitative data beyond surface meanings.^20^ A broad analytical framework for coding stigma-related issues within the dataset was established by OB, based on inductive coding of randomly selected transcripts and deductive categories derived from general stigma theory and specific concepts (anticipated stigma, public stigma, experienced stigma and self-stigma), as defined above.^1^ This was initially populated and adapted by site coordinators who coded their site's data with the aid of NVivo 10. OB then conducted a further layer of deductive coding on the superordinate stigma themes from each site to analyse them in respect of the aforementioned temporal perspectives. Ongoing data analysis, emerging hypotheses and the analytical approach were discussed with site coordinators during regular meetings.

## Results

### Stigma in everyday time

Across the sites, stigma within the daily routines and repetitive dilemmas of PLHIV's lives included: (1) the immediate *presence* of stigma in everyday situations, manifest as repeated gossiping, mocking and laughing, and (2) the relative *absence* of supportive interpersonal discourse, which fuelled information management and judicious disclosure.

The presence of stigma, across the sites, typically materialised in *anticipated* gossiping, though numerous examples of *experienced* gossiping existed too. While some PLHIV ignored community gossip and engaged in care, for others, its insidiousness influenced their termination or intermittence of treatment:

After the test I was told that I have the virus and I accepted it. I went back home and when we were bathing in the rivers I was sickly and they laughed at me. You see, this made me stop treatment. (Male, PLHIV, on ART, Zimbabwe)

Public stigma (occasionally enacted by healthcare workers) disrupted PLHIV's transitioning throughout the care and treatment continuum, but especially during testing and early counselling, and also initial ART adherence. Central to fear of gossip was the lack of privacy and confidentiality experienced daily at community clinics and hospitals.

How will they [neighbours] regard me being in the queue there, on the bench waiting for the medicines, or when I've gone for testing…how will they think of me? (Male, PLHIV, on ART, Tanzania)

Fear of gossiping and mocking prompted some PLHIV to retreat from clinics or travel long distances to those far away from their communities:

I come to a far away clinic, no one will know that I have come here to take drugs. (Female, PLHIV, on ART, Kenya)

### Stigma in biographical time

Biographical time frames everyday stigma events to highlight the dilemma of disclosure in relation to sexual relationship norms, as well as the interfacing of age and healthcare continuum points.

#### Disclosure and relationships

The dilemma of disclosure within—and its threat to—heterosexual and marital relations, especially for younger people, was vexed and salient. One young Ugandan woman recounted: ‘If you had no man, you wouldn't get anyone [if they knew your status]. If someone loved you, [your friends] would say to them, “that one is sick”’. This issue complicated couple-testing policies at each location.

They [pregnant women] are all accepting to get tested. We have a few problems where they do not want to get tested or after getting tested they do not want their results. If that person takes the results she might refuse to take treatment… this is mostly done by the young mothers who have recently got married. They are afraid to test positive and afraid to tell their husbands…afraid that the marriage might be broken. (Healthcare worker, Zimbabwe)

Within the sample, complete and ongoing concealment in interpersonal relationships was rare. Yet, disclosure was individual and time-sensitive, a biographical step-change that was risky across relationships. Potential social support from partners, friends, family and neighbours, often forthcoming consequent upon disclosure, simultaneously posed a risk of discrimination and blame:

After finding out that I was positive, and my sister in law scolding me, I saw that it was better someone dies rather than feeling the pain of being rudely talked to. (Female, PLHIV, on ART, Kenya)

That said, when some PLHIV did not disclose, they continued to hear negative comments from their loved ones about PLHIV, became alienated from other PLHIV and sometimes missed clinical appointments:

The person I stay with—my mother in law—I have not disclosed to her. So finding time to come here [clinic] is a bit difficult, so most of the times I come late, at times I miss coming. (Female, PLHIV, on ART, Kenya)

We still have a problem especially us young males, even me. Before I tested positive…I didn't like someone who is HIV positive. I remember when another guy disclosed his status, while we were busy eating meat, I chose to stop eating with them. What I used to see is people are talking badly about HIV even though they are HIV positive. Now I can see why they do that; they want to protect their names from being known that they are HIV positive. (Male, PLHIV, on ART, South Africa)

Family members tended to offer most support to PLHIV after disclosure, across the sites. However, some PLHIV only disclosed to partners, especially when there was family discord. Indeed, PLHIV sometimes concealed their diagnosis and treatment from their family members even after ART initiation.

#### Age and HIV care experience

The second way biographical time contextualised everyday stigma events was in respect of age and point along the care and treatment continuum. In this respect, gossiping was less affecting for those over 40 and those more stable on ART, perhaps because some goals (eg, courtship and reproduction) had already been achieved. That said, some older people were marginalised and considered already dead by their family members. A healthcare worker in Kisesa recounted an older woman's experience:

After further encouragement, her family members visited, they refused to be supportive and started to divide the old woman's possessions as she watched…They told her they will stay distant until she passes away then take her remaining possessions.

Moreover, some older people reported unease at clinics where they were often outnumbered by younger people whom, they imagined, speculated about their sexual activity:

We old people mix with children there [hospital]… people tend to think about how we old people got infected with this disease. (Male, PLHIV, on ART, Malawi)

### Stigma in epochal time

Epochal patriarchal relations gave a structural context to everyday and biographical stigma dynamics. Historical shifts to social acceptance of PLHIV within these communities, while positive, were complicated by stigma in everyday life and in respect of biographical goals like having a family. Moreover, low community-level resistance to HIV-related stigma, and contradictory strategies, such as integrating or separating patients with HIV in clinics, jeopardised and confused stigma reduction strategies.

#### Patriarchy

In combination with a reluctance to disclose, men were often able to mobilise patriarchal structures to avoid testing, whether alone or part of a couple.

Interviewer: Did you test together with your husband?

Participant: No, he does not allow, he does not accept. He does not tell me. I got this feeling that he is like me [HIV+] but fears to tell me. (Female, PLHIV, diagnosed not in care, Uganda)

Moreover, men often insisted that their partners get tested and disclose first, which they used to absolve themselves from any part in bringing HIV into relationships. HIV-positive women sometimes hid their status or stopped pill-taking for fear of domestic abuse or divorce. One young woman in Karonga, on ART while pregnant, hid her ARVs in a bag of flour, scared that her husband would have beaten her up or even killed her if he had discovered her status.

#### Normalisation?

Notwithstanding enduring issues of patriarchy, there were signs of increasing social acceptance of HIV over historical time within these communities. HIV was acknowledged as widespread, even ‘normal’, and no longer viewed uniformly as a death sentence in the age of ART; many participants were ‘not shocked’ at being diagnosed.

I just felt normal about having [AIDS], like it was a normal thing because it's not just me who has it…why would I be shocked? It has become a disease for everyone. (Female, PLHIV, on ART, Tanzania)

There were suggestions that marginalisation of HIV was decreasing across these communities. While any historical decline of marginalisation might seem positive, it runs in tandem with the persistence and power of everyday gossiping and laughing mentioned above.

Each household where there is person living with HIV, those people accept it, they understand now. But where there is no one with HIV they see it as a joke. (Female, PLHIV, Diagnosed but not on ART, Zimbabwe)

Indeed, some of participants' discourse suggested a hope for idealised situation rather than an experienced reality. Relatedly, some PLHIV who described this epochal shift to social acceptance simultaneously struggled with disclosure. Also, some healthcare workers went so far as to claim that ‘currently there is no stigma’ (Karonga) in their communities allowing people to test more freely. Yet, this view sometimes contrasted markedly with the daily experiences of PLHIV, shown above.

#### Challenging stigma?

Community-level challenges to stigma remain low and fragmented across all sites, suggesting epochal stasis, due to a lack of resources. Support most often came privately from immediate family and friends:

My husband, once I had told him… he was the one who advised me to just come and take drugs, that it was good. (Female, PLHIV, on ART, Kenya)

That said, some older PLHIV reported instances of community members being fined and sanctioned for gossiping (eg, in Malawi, Zimbabwe, Uganda), and there were a few examples of local opinion leaders organising well-attended public HIV discussions:

Church leaders, like priests and catechists, preached against community stigma in their communities. (Healthcare worker, Uganda)

In the current epoch, increased visibility of HIV-related activities in the community can have both positive and negative effects on stigma. For example, integrated healthcare services were considered by some PLHIV and healthcare workers as destigmatising in Kyamulibwa (because it reduced ‘othering’), but stigmatising in Kisesa and Karonga (because of lack of privacy).

We mixed HIV patients with general ones because of stigma. Patients were afraid of being seen coming from the counselling room. (Healthcare worker, Uganda)

## Discussion

Within these communities, HIV-related stigma persists in everyday, biographical and epochal time. These temporal framings shed light on why PLHIV cycle in and out of care,^21^ why the 2020 UN stigma reduction target is ambitious^2^ and why stigma persists in the age of ART.^7^

*Everyday* stigma events included gossiping, laughing and mocking from community members, and relatively absent supportive community HIV-related discourse. This contributed to delayed uptake of testing^7^ ^12^ and intermittent postdiagnosis care engagement, supporting findings from other recent single-country^15^ ^17^ ^22^ and multicountry^23^ ^24^ analyses. Continued public concealment of seropositivity, especially during earlier stages of care, influences the absence of tolerant discourse. However, few PLHIV disclosed to no one, as has been observed elsewhere.^24^ The absence of constructive discussion of HIV in public spheres has previously been reported by Campbell *et al*^25^ in South Africa. When gossip is the main presence of HIV discourse in public life, it restricts the daily support for PLHIV. Moreover, persisting forms of everyday stigma frustrate the biographical goal of HIV stabilisation and the epochal one of AIDS elimination. Private support within families and friendships has enabled greater acceptance, but this needs to translate into everyday advocacy and accepting discourses in public too.^26^ Healthcare staff, policy-makers and community leaders should help PLHIV understand everyday experiences of stigma in light of wider epochal shifts to social acceptance; where PLHIV are unable to detect these changes in their everyday lifeworlds, then their care engagement may be undermined.

*Biographical* time, especially in respect of pressures to partake in socially and sexually important projects and practices, contextualises why everyday stigma events are important throughout the life course and at different points along the care and treatment continuum, particularly during testing and ART adherence. Healthcare workers and policy-makers must recognise the importance of HIV-related stigma to biographical life goals, such as sexual relationships and childrearing, and work with PLHIV and their partners to find accepting solutions.

The finding that HIV-related stigma is affected by *epochally* persisting gender and socioeconomic inequalities, within these communities, reflects earlier studies^17^ ^22^ and indicates the importance of challenging structural stigma in respect of enduring relations of poverty. It should be acknowledged that disclosure in patriarchal and sexist contexts may not be of benefit to PLHIV.^27^ Interventions need to be targeted at gender structures^5^ and different age groups throughout the life course and specific community contexts. Our findings also mirror those of Church *et al*^28^ who noted the complex relationship between models of HIV care (stand-alone or integrated) and stigma; practical guidance for healthcare workers to minimise stigma across the different models is therefore needed. Notwithstanding the effect of these epochally entrenched and complex conditions on HIV-related stigma, it appears a combination of successful health education, improvements to health services and the provision of ART^9^ has partially tackled HIV-related stigma and helped normalise HIV.^15^ To go further, there is a great need for village leaders and clinical staff to be supported—financially, legally and normatively—to innovate new ways of generating community discussion and informing policy. Moreover, HIV healthcare should be delivered through flexible community outreach, and engagement techniques should be developed for those who may avoid such visits for fear of being exposed as HIV-positive to neighbours. To insist that PLHIV attend clinics to obtain treatment is to fail to acknowledge the importance of stigma in everyday, biographical and epochal time: the consequence can be that PLHIV wait until they are desperate or die before they get help.

Intractable epochal structures that facilitate stigma, or make no inroads into its mitigation, need to be tackled by a concerted collective effort, which policy-makers need to be part of. Central to this will be investment in community, national and international interventions to destigmatise. As social contact interventions, in the context of improving HIV-related education, appear to offer optimistic avenues for HIV-related stigma reduction,^29–31^ there is a need to continue health education efforts and create considered public interactions between those infected and those who are not across these communities.

Our study has several strengths and limitations. Experienced, local research teams and access to HDSS databases enhanced recruitment and data collection of PLHIV, especially those who were no longer in care. While the generalisability of our findings is limited by the small sample size, cross-country analysis showed a considerable commonality across the research sites, which may be generalisable to other rural populations in sub-Saharan Africa. Participants had some difficulty discussing sensitive issues and experiences; however, this was minimised by ensuring confidentiality and by using trained, local interviewers of different genders and ages who avoided judgemental reactions. People experiencing most extreme or violent forms of stigmatisation were perhaps most likely to be under-represented in the study, for example, those lost to follow-up.

## Conclusion

Future HIV-related stigma research would benefit from being sensitive to temporality. Consideration of this allows a multifaceted view of HIV-related stigma to emerge throughout the care and treatment continuum, across epidemics. Stigma certainly disrupts testing, but it can also lead to intermitting or terminating postdiagnosis care. In light of historical, biographical and daily persistence of stigma events, there is an urgent need to develop tailored healthcare plans for PLHIV, and for greater investment in stigma mitigation.

**Key messages**

- A novel temporal framing of HIV-related stigma dynamics can usefully situate and interpret experiences of people living with HIV (PLHIV) across the HIV care continuum.
- *Everyday* time frames HIV-related stigma to show the repetitive *presence* of gossiping and mocking, but also the relative quotidian *absence* of supportive interpersonal discourse in public life across these communities.
- *Biographical* time frames everyday stigma events, highlighting the dilemma of disclosure in relation to sexual relationship norms, as well as the interfacing of age and healthcare continuum points.
- *Epochal* time shows the importance of persisting patriarchy to stigma experience. There are, however, signs of increasing social acceptance of PLHIV over historical time within these communities.
- There is a great need to focus resources on multilevel resistance to HIV-related stigma that takes account of imbricated temporalities.
